# Supplementary material for: Taxifolin and Lucidin as Potential E6 Protein Inhibitors: p53 Function Re-Establishment and Apoptosis Induction in Cervical Cancer Cells
Source: Cancers (Basel). 2022 Jun 8;14(12):2834. doi: 10.3390/cancers14122834 (PMC9221127; doi:10.3390/cancers14122834)
Supplement: Supplementary file 1 [file cancers-14-02834-s001.zip › cancers-1730912-supplementary.pdf]

Supplementary Materials: Taxifolin and Lucidin as Potential E6 Protein Inhibitors: p53 Function Re-Establishment and Apoptosis Induction in Cervical Cancer Cells

Diana Gomes, Shivani Yaduvanshi, Samuel Silvestre, Ana Paula Duarte, Adriana O. Santos, Christiane P. Soares, Veerendra Kumar, Luís Passarinha and Ângela Sousa

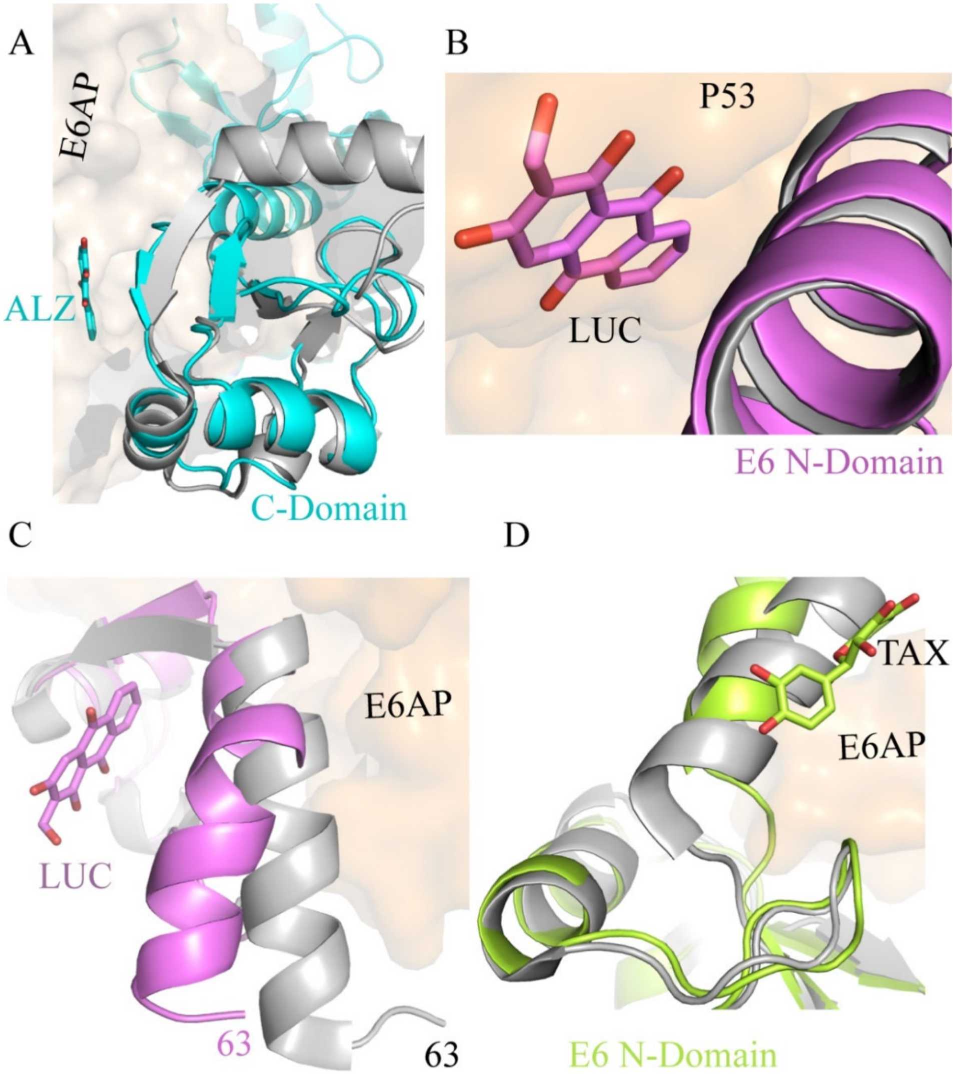

Figure S1. The phenolic compounds alizarin (ALZ), lucidin (LUC) and taxifolin (TAX) might inhibit the binding of E6AP and p53 on E6. Superimposition of ternary complex E6/E6AP/p53 on simulated E6 structure from A) E6/alizarin complex, B), C) E6/lucidin complex, and D) E6/taxifolin complex. The Phenolic compounds slip into interface of E6 and p53/E6AP, thereby, destabilize the complex. The phenolics compounds are at the interface. E6 before simulation (grey) and after simulation (coloured) is shown as cartoon. The p53/E6AP are shown in surface representation.

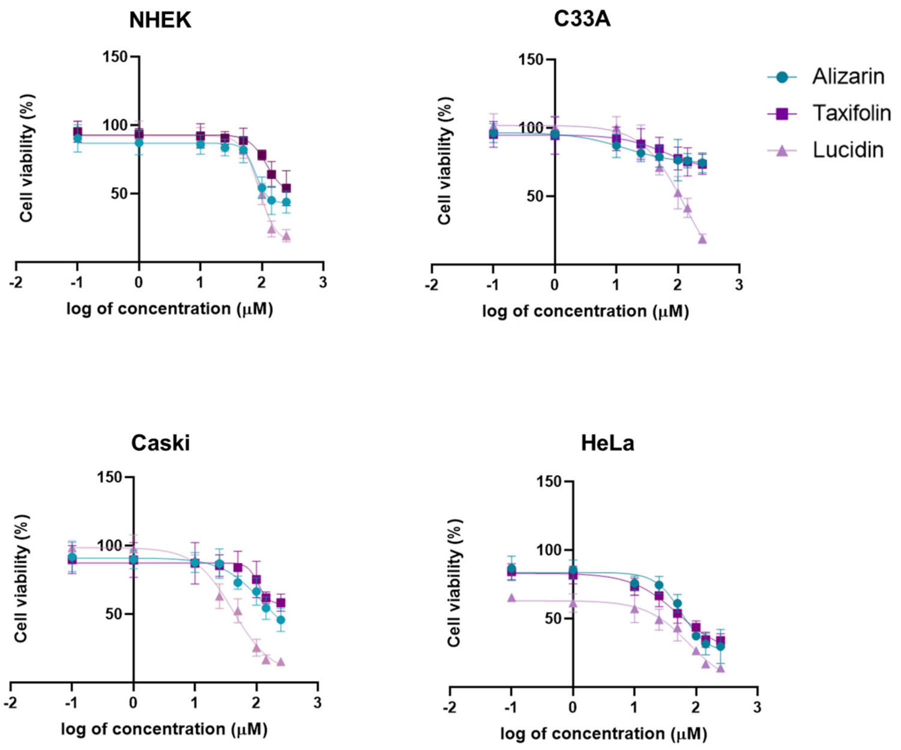

Figure S2. Dose-response curves for alizarin, taxifolin and lucidin in NHEK, C33A, Caski and HeLa cell lines.

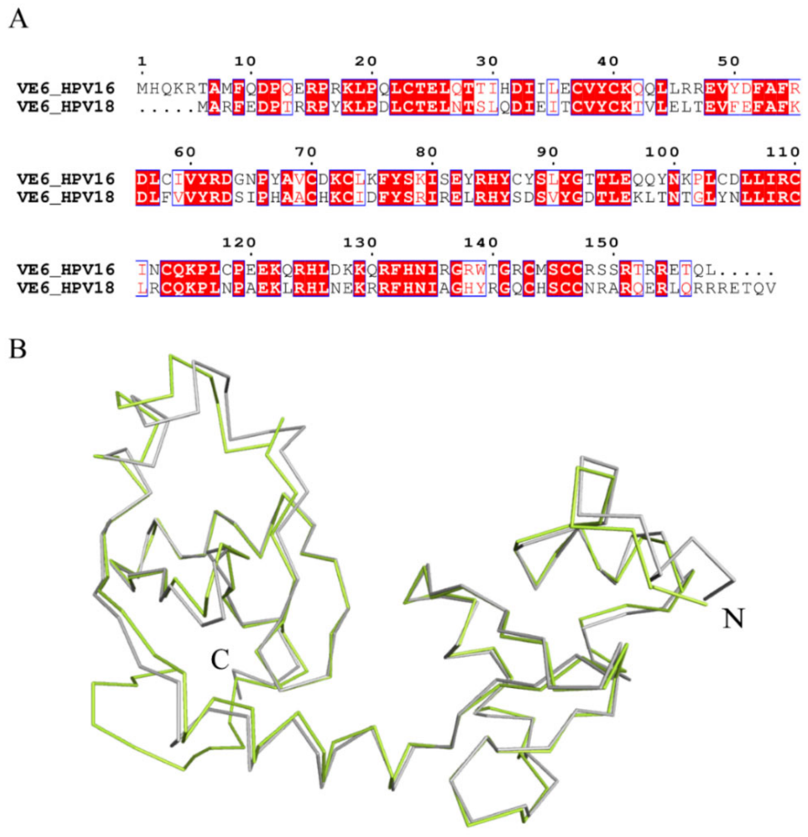

Figure S3. Comparison of E6 protein from HPV-16 and HPV-18. A) Sequence alignment of 16E6 and 18E6. Similar residues are shown in red, whereas identical residues are highlighted as white letters

on a red background. This diagram was prepared using the program ESPript (https://espript.ibcp.fr/ESPript/ESPript/). B) The overall structure of 16E6 (limon) and 18E6 (grey) is very similar.

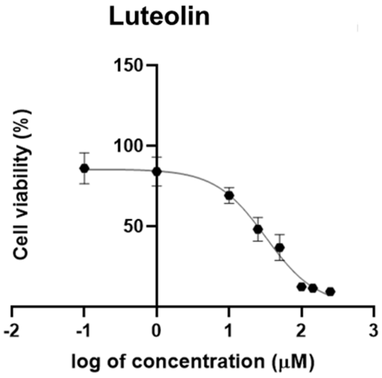

Figure S4. Dose-response curve for luteolin in HeLa cell line.

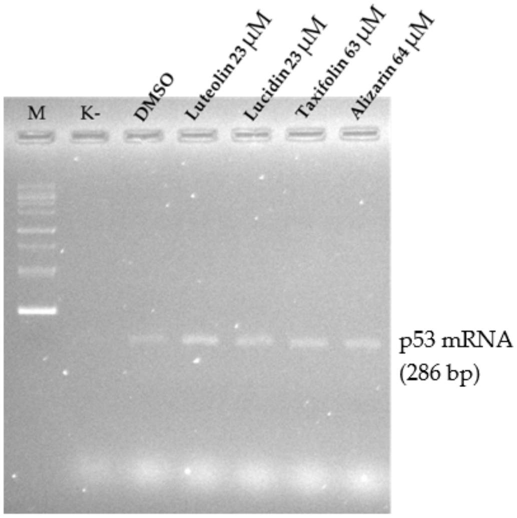

Figure S5. The effect of phenolic compounds on p53 mRNA levels in HPV18 cells was evaluated by RT-PCR analysis after 24 h of treatment. M: DNA ladder and K$^-$: negative control of PCR.

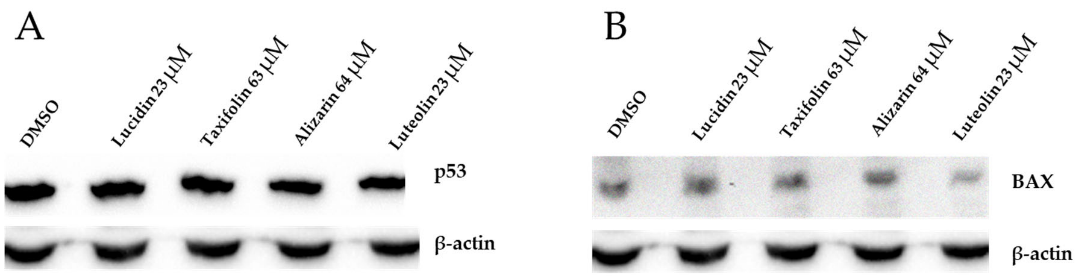

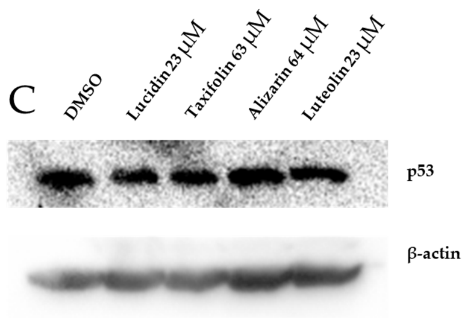

Figure S6. The effect of anthraquinones and flavonoids on p53 (A) and BAX (B) protein of HPV negative C33A cells and the effect on p53 protein of NHEK cells (C) was evaluated by Western blot analysis after 48 h of treatment.

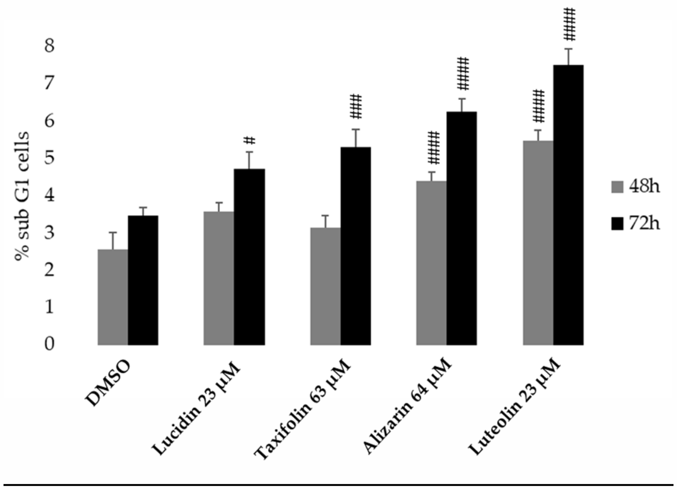

Figure S7. Percentage of subG1 cells obtained by flow cytometry of HeLa cells treated with phenolic compounds for 48h and 72h. The analysis was performed by *t*-Student test (statistical significance determined as p-values #<0.05, ##<0.01, ###<0.001, ####<0.0001 in comparison with the respective DMSO-treated cells).

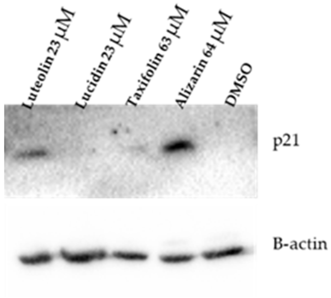

Figure S8. The effect of anthraquinones and flavonoids on p21 protein levels in HPV positive Hela cells was evaluated by Western blot analysis after 48 h of treatment.

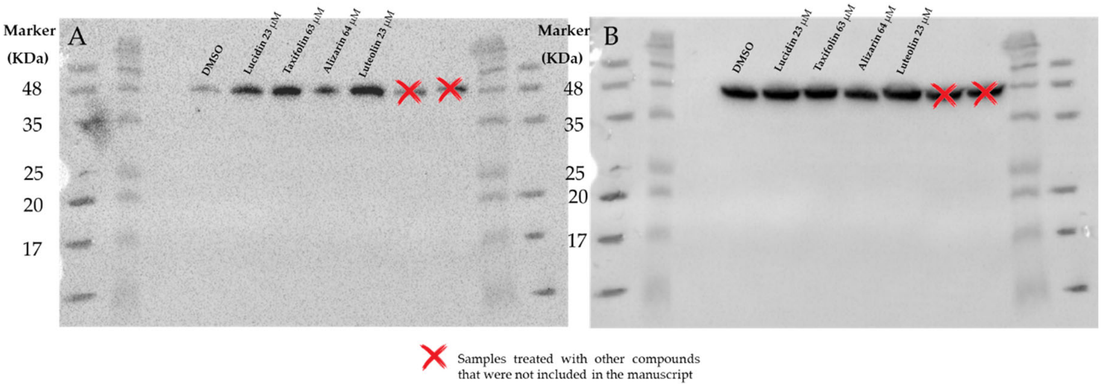

Figure S9. Uncropped blot of figure 6A. A – p53 protein and B – β-actin protein. .

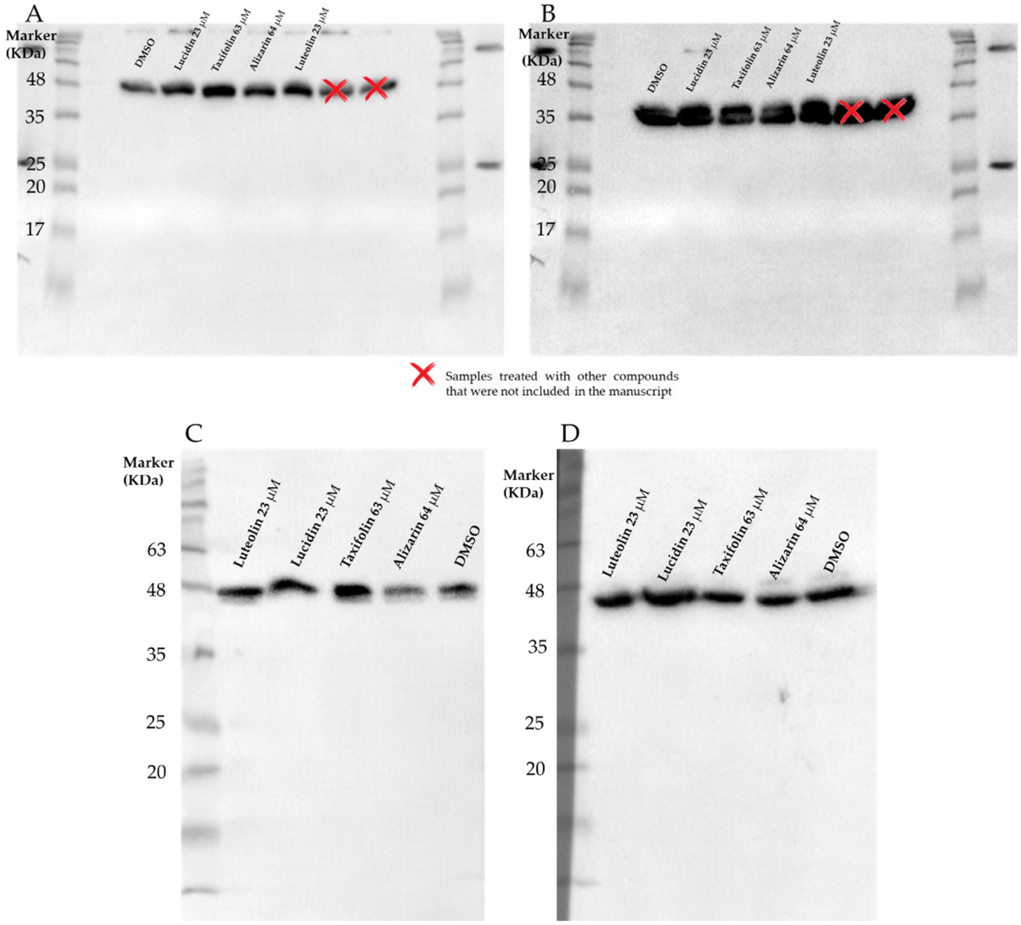

Figure S10. Uncropped Blot duplicates of figure 6A. A, C – p53 protein and B, D – β-actin protein. .

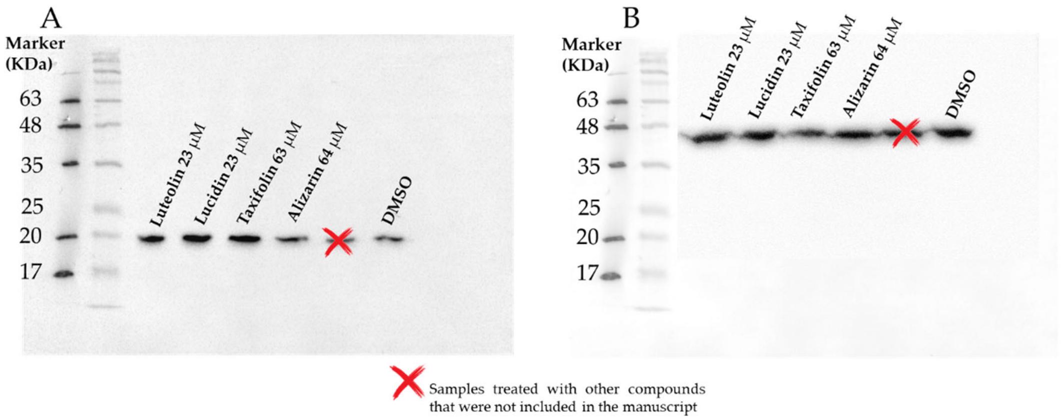

Figure S11. Uncropped blot of figure 6B. A – BAX protein and B – β-actin protein. .

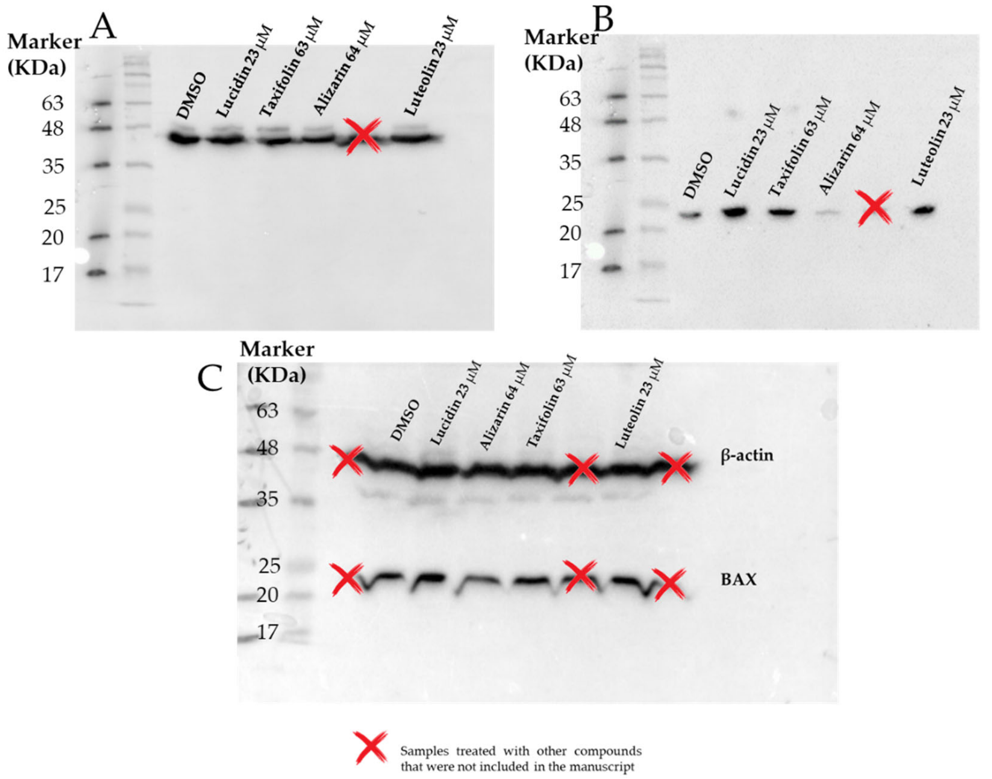

Figure S12. Uncropped Blot duplicates of figure 6B. A – BAX protein, B– β-actin protein and C – BAX and β-actin proteins. .

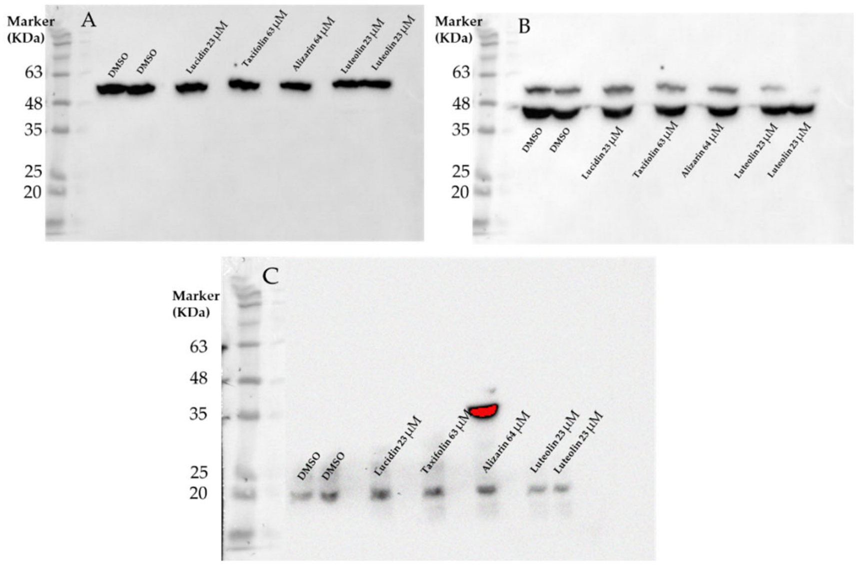

Figure S13. Uncropped blot of figure S6A e S6B. A – p53 protein, B – β-actin protein and C – BAX protein. .

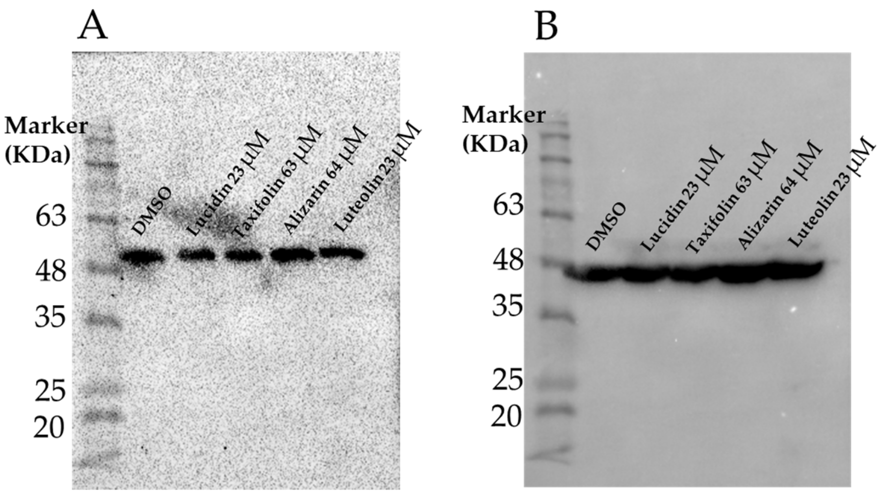

Figure S14. Uncropped blot of figure S6C. A – p53 protein and B – β-actin protein.

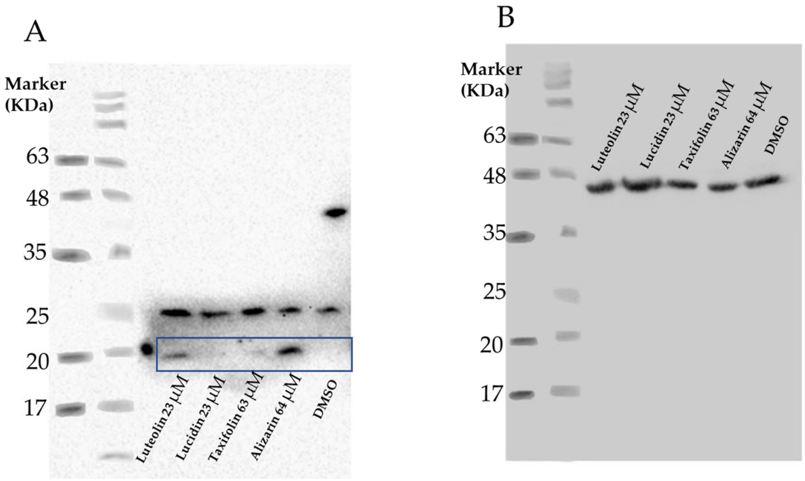

Figure S15. Uncropped blot of figure S8. A – p21 protein and B – β-actin protein.
